# Exploring the Effectiveness and Safety of Azilsartan-Medoxomil/Chlorthalidone Versus Olmesartan-Medoxomil/Hydrochlorothiazide in Hypertensive Patients: A Meta-Analysis

**DOI:** 10.7759/cureus.41198

**Published:** 2023-06-30

**Authors:** Lakshya Kumar, Sundal khuwaja, Aanand Kumar, Unaib Ahmed Memon, Munesh Kumar, Arpana Ashok, Manisha lohana, Ahmed Qudoos, Maham kashif, Mahima Khatri, Satesh Kumar, FNU Sapna, Arjan Dass, Giustino Varrassi

**Affiliations:** 1 General Medicine, Pandit Dindayal Upadhyay (PDU) Medical College, Rajkot, IND; 2 Medicine, Liaquat University of Medical and Health Sciences, Jamshoro, PAK; 3 Internal Medicine, Liaquat University of Medical and Health Sciences, Jamshoro, PAK; 4 Department of Medicine, Jinnah Postgraduate Medical Centre, Karachi, PAK; 5 Medicine, Khawaja Muhammad Safdar Medical College, Sialkot, PAK; 6 Medicine and Surgery, Dow University of Health Sciences, Karachi, PAK; 7 Medicine and Surgery, Shaheed Mohtarma Benazir Bhutto Medical College, Karachi, PAK; 8 Internal Medicine, Detroit Medical Center, Detroit, USA; 9 Internal Medicine, Willis-Knighton Health System, Shreveport, USA; 10 Pain Medicine, Paolo Procacci Foundation, Rome, ITA

**Keywords:** systematic review, meta-analysis, azilsartan-medoxomil, olm/hctz, azi-m/ct, olmesartan, chlorthalidone

## Abstract

This study aims to assess the effectiveness and safety of azilsartan-medoxomil/chlorthalidone (AZI-M/CT) compared to olmesartan-medoxomil/hydrochlorothiazide (OLM/HCTZ) in patients with hypertension. Systematic searches were conducted on PubMed, Google Scholar, and ClinicalTrials.gov, starting from their establishment until March 15, 2023. The purpose of these searches was to locate original reports that compare the effectiveness of AZI-M/CT and OLM/HCTZ in treating hypertension. Data on various characteristics at the beginning and end of the studies were gathered. The analyses were carried out using Review Manager 5.4.1 (The Nordic Cochrane Center, The Cochrane Collaboration, 2014, Odense, Denmark) and STATA 16.0 software (Stata Corp. LP, College Station, TX, USA). Risk ratios (RRs) and weighted mean differences (WMDs) with 95% confidence intervals (CIs) were calculated as part of the study. A total of 3,146 individuals from four separate investigations were included in the study, with 1,931 individuals receiving AZI-M/CT and 1,215 individuals receiving OLM/HCTZ. The combined analysis revealed that the average diastolic blood pressure (DBP) was significantly lower in the AZI-M/CT group compared to the OLM/HCTZ group (WMD -2.64 [-2.78, -2.51]; *P *= 0.00001; *I*^2^ = 1%). However, there were no significant differences in mean systolic blood pressure (SBP; WMD -2.95 [-6.64, 0.73]; *P *= 0). Furthermore, the AZI-M/CT group had a notably higher incidence of major adverse events (RR 1.58 [1.20, 2.08]; *P *= 0.001; *I*^2^ = 11%) and any treatment-emergent adverse events (RR 1.11 [1.03, 1.20]; *P *= 0.007; *I*^2^ = 51%). However, there was no significant difference in the mortality risk between the two groups (RR 0.74 [0.14, 3.91]; *P *= 0.72; *I*^2^ = 0%). Based on the results of our meta-analysis, AZI-M/CT is more effective than OLM/HCTZ at reducing blood pressure in elderly hypertensive patients. However, because of the small sample size, favorable results must be carefully reevaluated, and more studies are needed.

## Introduction and background

Hypertension is characterized by systolic blood pressure exceeding 140 mm Hg and diastolic blood pressure exceeding 90 mm Hg. It is strongly linked to cardiovascular morbidity and mortality. A study conducted in 2013 revealed that systolic hypertension was responsible for approximately 10.4 million deaths worldwide [1]. Consequently, the European Hypertension Society has recommended lifestyle changes and pharmacological treatment for managing hypertension [2]. Angiotensin receptor blockers (ARBs) and thiazide diuretics are commonly prescribed among the pharmaceutical therapies considered. ARBs and thiazides are the preferred initial treatments for hypertension due to their ability to lower blood pressure [3]. These drugs also offer protective benefits by reducing life-threatening events like heart failure and stroke [1,4].

Azilsartan-medoxomil (AZI-M) is a recently approved long-acting ARB with greater potency and effectiveness than other ARBs [5]. While thiazide diuretics are the first-line treatment, there is an ongoing debate about the comparative effects of hydrochlorothiazide (HCTZ) and chlorthalidone (CT) [6]. Both medications demonstrate preventive and beneficial impacts in reducing high blood pressure and the risk of cardiovascular events. These can be used alone or in combination. However, it is essential to note that these medications also have adverse side effects [4]. These include electrolyte imbalances, fluctuations in blood glucose levels, renal complications, and worsening gout symptoms.

Numerous comparative studies have analyzed different drug categories within and between groups. These investigations have comprehensively overviewed the relative efficacy and safety profiles [7]. However, current studies have yielded varied outcomes due to the heterogeneity of the population and insufficient sample sizes to establish conclusive associations. To address this, we conducted a meta-analysis of the latest findings to comprehensively examine the blood pressure-lowering effects of AZI-M/CT in combination with HCTZ compared to olmesartan (OLM) in conjunction with HCTZ.

## Review

Methods

Methodology

The methods used in this meta-analysis adhere to the guidelines and criteria set forth by the Preferred Reporting Items for Systematic Review and Meta-Analysis (PRISMA) [8,9].

Search Strategy and Selection

A comprehensive and systematic scientific literature search was performed until March 15, 2023, on three databases: PubMed, Google Scholar, and ClinicalTrials.gov. The search terms used included relevant subject keywords and their MeSH terms, namely, (efficacy OR tolerability OR safety) AND (azilsartan OR ARB OR Angiotensin receptor blocker OR medoxomil) AND (Chlorthalidone OR thiazides) AND (Olmesartan) AND (hydrochlorothiazide) AND (chronic kidney disease OR chronic renal disease). Two reviewers (SK and MK) independently filtered the search results, and any discrepancies were resolved through consultation with a third reviewer (LK). Initially, studies were selected based on the title and abstract. Subsequently, the full text of eligible studies was assessed for inclusion. Furthermore, the references of selected papers were thoroughly examined for additional relevant studies.

Study Inclusion and Exclusion criteria

The studies included in this analysis met specific criteria for eligibility, as outlined below: They were published entirely in the English language, they involved patients diagnosed with hypertension, and they evaluated the effects of AZI-M/CT on relevant outcomes in hypertensive patients and compared them to the effects of OLM/HCTZ. Furthermore, these studies were assessed to ensure that they provided essential information on each drug group's efficacy and side effect profiles separately. Review reviews, editorials, procedures, case reports, and studies that lacked a comparison or results section were excluded from consideration.

Data Extraction

Key information was extracted from the relevant publications, including the primary author, publication year, trial type, and phase, as well as the duration of the study follow-up. Details regarding the dosages of the administered drugs, the total number of patients included in the study, and the patient counts for each group (AZI-M/CT and OLM/HCTZ) were also collected. Additionally, the supplementary antihypertensive medications and baseline parameters were retrieved. Several primary outcomes were identified and extracted, including mortality, treatment-emergent adverse events (TEAEs), serious adverse events, the severity of adverse events, the number of patients titrated to a higher dose, mean blood pressure, the number of patients who achieved the target blood pressure, system-associated adverse events, and changes in laboratory parameters.

Assessment of Risk of Bias

The quality of all randomized controlled trials (RCTs) was assessed using the risk-of-bias assessment tool developed by the Cochrane Collaboration [8].

Data Analysis

Statistical analysis was performed using Review Manager 5.4.1 (The Nordic Cochrane Center, The Cochrane Collaboration, 2014, Odense, Denmark) and Stata 16.0 (Stata Corp. LP, College Station, TX, USA). The raw data from the included studies were utilized to calculate the relative risks (RRs) for dichotomous data and the weighted mean differences (WMDs) for continuous data, accompanied by 95% confidence intervals (CIs). These measures were pooled using a random-effects model. The findings of the pooled studies were presented through forest plots. To assess publication bias, Begg's test and funnel plots were employed for effectiveness outcomes, TEAEs, and major adverse events. Heterogeneity was evaluated and categorized as low (≤25%), moderate (25%-75%), or high (>75%) using Higgin's *I*^2^ tests [10]. A *P*-value of 0.05 was considered significant for all analyses.

Results

**Figure 1:**
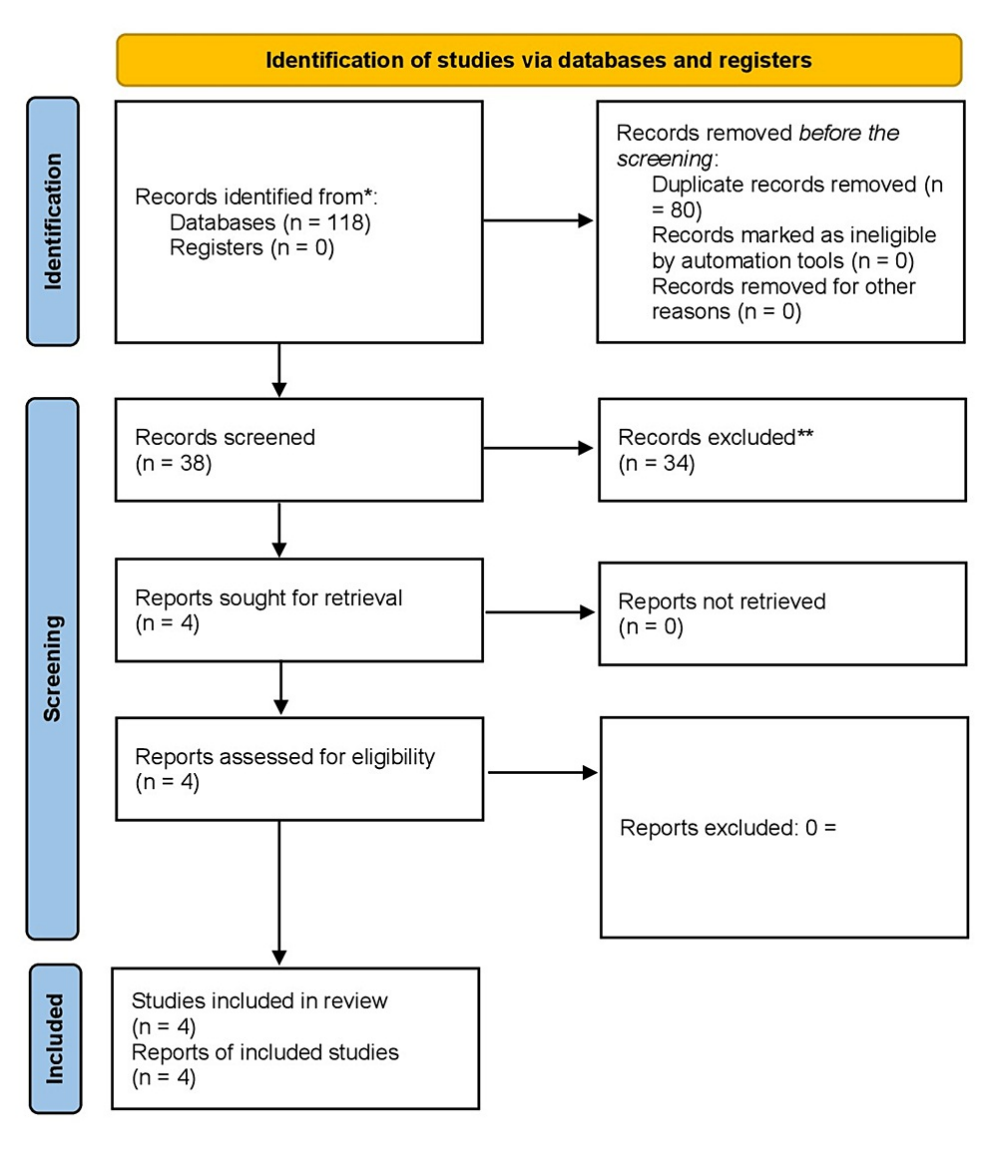
PRISMA flow diagram illustrating the search strategy and study selection process for the meta-analysis. PRISMA, Preferred Reporting Items for Systematic Review and Meta-Analysis

Initially, a comprehensive search of the relevant literature yielded 118 articles. Four RCTs [5,11-13] were included in this meta-analysis by excluding duplicate articles and evaluating titles and abstracts. The selection process is visually represented in Figure 1, which follows the PRISMA flowchart and provides a clear overview of the comprehensive inquiry.

Characteristics of Participants

The total number of individuals included in the analyzed investigations was 3,146: 1,931 (61.3%) individuals received AZI-M/CT, while 1,215 (38.6%) individuals were assigned to the OLM/HCTZ group.

Tables 1 and 2 present a comprehensive overview of patients' characteristics based on the respective treatment modalities. Most patients were male (*n* = 1,742, 55.37%) and of Caucasian ethnicity (*n* = 2,263, 72%). Their mean age was 59.7 +/- 10.02. Among the patients in the AZI-M/CT group, the mean body mass index (BMI) was 31.28 +/- 6.2; in the OLM/HCTZ group, it was 31.75 +/- 6.3. At the beginning of the study, diabetes was present in 325 patients receiving AZI-M/CT and 227 patients receiving OLM/HCTZ (16.8% and 18.6%, respectively). The mean systolic blood pressure (SBP) was 158.59 +/- 10.7 in the AZI-M/CT group and 157.62 +/- 10.2 in the OLM/HCTZ group. The mean diastolic blood pressure (DBP) was 91 +/- 10.2 overall and 90.9 +/- 10.18 in the AZI-M/CT group. Additionally, a majority of hospitalized patients had an estimated glomerular filtration rate (eGFR) ranging from 60 to 90 mL/minute/1.73 m², with 943 patients (48.8%) in the AZI-M/CT group and 518 patients (42.16%) in the OLM/HCTZ group falling into this range.

**Table 1:** Baseline demographics of patients. AZI-M/CT, azilsartan-medoxomil/chlorthalidone; OLM/HCTZ, olmesartan-medoxomil/hydrochlorothiazide; *n*,  number of patients; N/A, not available; SD, standard deviation; RCT, randomized controlled trial

Study and year	Study design	Phase of trial	Total number of participants	Duration of study	Patients in AZI-M/CT	Patients in OLM/HCTZ	Dose of drug (mg/day)		Age (years), mean (SD)		Male gender, *n* (%)		Prior antihypertensive use, *n* (%)	
							AZI-M/CT	OLM/HCTZ	AZI-M/CT	OLM/HCTZ	AZI-M/CT	OLM/HCTZ	AZI-M/CT	OLM/HCTZ
Cushmann et al. (2012) [5]	RCT	3	1071	12 weeks	707	364	40/25,80/25	40/25	56.4 (10.5)	56.7 (10.1)	424 (59)	205 (56)	554 (78.35)	277 (76.1)
Neutel et al. (2017) [11]	RCT	3	837	52 weeks	418	419	40/12.5,80/12.5,809/25	20/12.5,20/25, 40/12.5, 40/25	58.5 (10.8)	57.6 (10.8)	226 (54)	247 (59)	N/A	N/A
Cushmann et al. (2018) [12]	RCT	3	1085	8 weeks	729	356	40/25, 40/12.5, 80/25	20/12.5, 40/25	56.1 (10.6)	55.7 (9.8)	380 (52.1)	183 (51.4)	N/A	N/A
Bakris et al. (2018) [13]	RCT	3	153	52 weeks	77	76	20/12.5, 40/12.5, 40/25	20/12.5, 40/12.5, 40/25	67.9 (8.24)	68.9 (9.1)	31 (41)	45 (59)	N/A	N/A

**Table 2:** Comorbidities of the patients included in the study. CKD, chronic kidney disease; SBP, systolic blood pressure; DBP, diastolic blood pressure; BMI, body mass index; AZI-M/CT, azilsartan-medoxomil/chlorthalidone; OLM/HCTZ, olmesartan-medoxomil/hydrochlorothiazide; *n*, number of patients; SD, standard deviation; N/A, not available

Study and year	Diabetes, *n* (%)		CKD , *n* (%)		Mean SBP (SD)		Mean DBP (SD)		Mean BMI (SD)	
	AZI-M/CT	OLM/HCTZ	AZI-M/CT	OLM/HCTZ	AZI-M/CT	OLM/HCTZ	AZI-M/CT	OLM/HCTZ	AZI-M/CT	OLM/HCTZ
Cushmann et al. (2012) [5]	113 (32)	65 (17.9)	N/A	N/A	150 (13.7)	149 (14)	88.25 (10.9)	87.1 (11)	31.65 (6.27)	31.6 (5.92)
Neutel et al. (2017) [11]	62 (15)	59 (14)	58 (14)	50 (12)	168 (7)	167.6 (7)	95.7 (9.2)	95.7 (9.6)	31.4 (6.2)	31.9 (6.6)
Cushmann et al. (2018) [12]	117 (16)	71 (19.9)	66 (9.1)	66 (9.1)	165 (10.6)	164.7 (10.4)	95.3 (10.25)	96.1 (10.4)	31.7 (6.1)	31.9 (6.1)
Bakris et al. (2018) [13]	33 (42.9)	32 (42.1)	N/A	N/A	151.1 (10.3)	149 (7.8)	84.8 (10.31)	84.7 (9.68)	30.4 (6.23)	31.6 (6.52)

Quality Assessment and Publication bias

**Figure 2:**
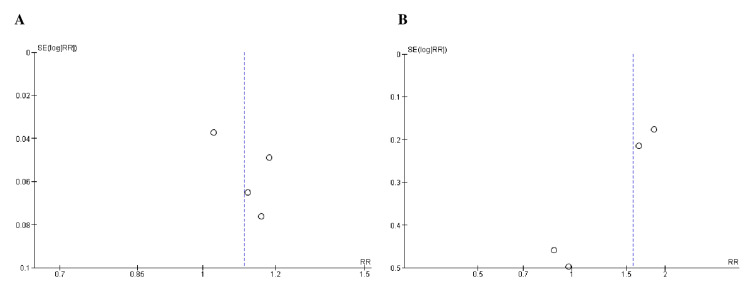
Funnel plots of safety outcomes: (A) any TEAE and (B) serious adverse event. RR was used as an effect measure and SE as a measure of precision. RR, relative risk; SE, standard error; TEAE, treatment-emergent adverse event

**Figure 3:**
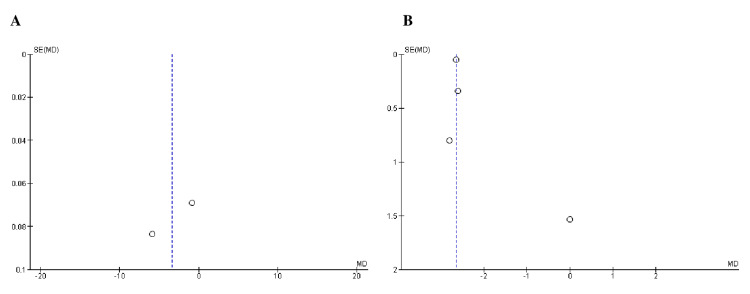
Funnel plots of efficacy outcomes: (A) mean SBP and (B) mean DBP. MD was used as an effect measure and SE as a measure of precision. MD, mean difference; SE, standard error; SBP, systolic blood pressure; DBP, diastolic blood pressure

**Figure 4:**
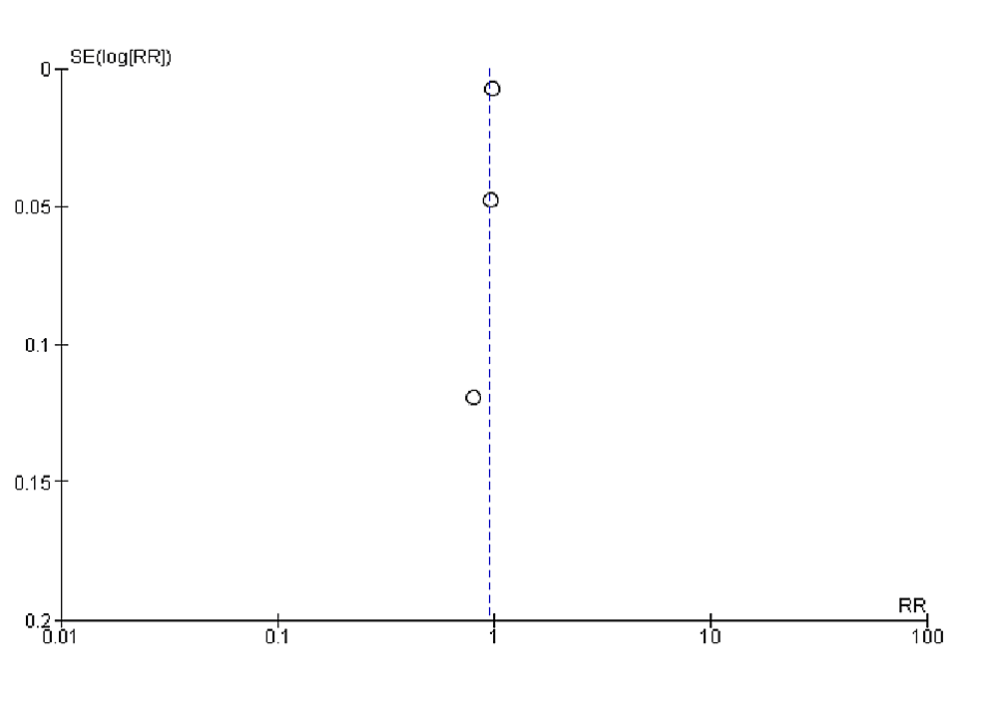
Funnel plot of achievement of target blood pressure. RR was used as an effect measure and SE as a measure of precision. RR, relative risk; SE, standard error

All studies included in the assessment of study quality using Cochrane's risk-of-bias tool demonstrated a low risk of bias, as indicated in Table 3. Furthermore, Begg's test (Table 4) and the funnel plots (Figures 2-4) showed no publication bias.

**Table 3:** Quality assessment of randomized controlled trials by Cochrane’s risk-of-bias tool.

Article	Selection bias		Performance bias	Detection bias	Attrition bias	Reporting bias	Other bias	Our evaluation
	Random sequence generation	Allocation concealment	Blinding of participants and personnel	Blinding of outcome assessment	Incomplete outcome data	Selective Reporting	Anything else, ideally prespecified	
Cushmann et al. (2012) [5]	Low risk	Low risk	Low risk	Low risk	Low risk	Low risk	Low risk	Good quality
Neutel et al. (2017) [11]	Low risk	High risk	High risk	High risk	Low risk	Low risk	Low risk	Poor quality
Cushmann et al. (2018) [12]	Low risk	Low risk	Low risk	Low risk	Low risk	Low risk	Low risk	Good quality
Bakris et al. (2018) [13]	Low risk	High risk	High risk	High risk	Low risk	Low risk	Low risk	Poor quality

**Table 4:** Begg’s test of efficacy outcomes and adverse events. TEAE, treatment-emergent adverse event; SBP, systolic blood pressure; DBP, diastolic blood pressure

Outcomes	Begg's test
Any TEAE	0.7341
Serious adverse event	1.9106
Mean SBP	1.2659
Mean DBP	0.3082
Achievement of target blood pressure	1.9633

Outcomes

An overview of patients’ outcomes by interventions is presented in Tables 5-7.

**Table 5:** Primary outcomes data extracted from included studies. AZI-M/CT, azilsartan-medoxomil/chlorthalidone; OLM/HCTZ, olmesartan-medoxomil/hydrochlorothiazide; TEAE, treatment-emergent adverse events; SBP, systolic blood pressure; DBP, diastolic blood pressure; *n*, number of patients; SD, standard deviation

Study and year	Total number of patients		Any TEAE (n)		Serious adverse events (*n*)		Death (*n*)		Mean SBP (SD)		Mean DBP (SD)		Achievement of target blood pressure (*n*)		Patients who were titrated to a higher dose *(n*)	
	AZI- M/C T	OL M/H CTZ	AZI- M/CT	OLM/ HCTZ	AZI- M/CT	OLM/ HCTZ	AZI- M/CT	OLM/H CTZ	AZI- M/CT	OLM/H CTZ	AZI- M/CT	OLM/ HCTZ	AZI- M/CT	OLM/ HCTZ	AZI- M/CT	OLM/ HCTZ
Cushmann et al. (2012)[5]	707	364	502	219	83	26	-	-	121.65 (0.94)	122.5 (1.13)	68.55 (0.86)	71.2 (0.8)	446	238	-	-
Neutel et al. (2017)[11]	418	419	328	320	79	43	2	2	125 (11.2)	129.6 (11.13)	77.3 (9.1)	80.1 (13.71)	-	-	-	-
Cushmann et al. (2018) [12]	729	356	392	171	12	6	-	-	125.7 (1.31)	131.6 (1.28)	76.3 (1)	78.9 (1)	713	353	266	184
Bakris et al. (2018) [13]	77	76	68	58	8	9	0	1	127 (17.88)	126 (17.17)	76 (9.6)	76 (9.25)	45	55	-	

 

**Table 6:** Adverse events. AZI-M/CT, azilsartan-medoxomil/chlorthalidone; OLM/HCTZ, olmesartan-medoxomil/hydrochlorothiazide; *n*, number of patients

Study and year	Hypotension (*n*)		Dizziness (*n*)		Headache (*n*)		Diarrhea (*n*)		Fatigue (*n*)		Myocardial infraction (*n*)		Cardiac arrest (*n*)		Pharyngitis (*n*)	
	AZI- M/C T	OL M/H CTZ	AZI- M/CT	OLM/ HCTZ	AZI- M/CT	OLM/ HCTZ	AZI- M/CT	OLM/H CTZ	AZI- M/CT	OLM/H CTZ	AZI- M/CT	OLM/ HCTZ	AZI- M/CT	OLM/ HCTZ	AZI- M/CT	OLM/ HCTZ
Cushmann et al. (2012) [5]	-	--	99	29	32	26	-	-	47	16	-	-	-	-	-	-
Neutel et al. (2017)[11]	-	-	68	53	31	46	-	-	21	17	0	1	1	0	-	-
Cushmann et al. (2018)[12]	7	1	49	20	28	18	27	5	21	5	-	-	-	-	-	-
Bakris et al. (2018)[13]	4	3	6	5	8	2	1	4	3	4	-	-	-	-	0	4

**Table 7:** Laboratory parameters. AZI-M/CT, azilsartan-medoxomil/chlorthalidone; OLM/HCTZ, olmesartan-medoxomil/hydrochlorothiazide; *n*, number of patients; SD, standard deviation; ULN, upper limit of normal

Study and year	Creatinine: two consecutive elevations (1.5 baseline and >ULN) (*n*)		Cr increased (*n*)		Mean fasting glucose (SD)		Fasting glucose shift from <7.0 to ≥7 mmol/L (*n*)		Shift from ≥7 to <7 mmol/L (*n*)		Hyperkalemia (*n*)		Hypokalemia (*n*)		Hyperuricemia (*n*)		Sodium from normal to low (*n*)	
	AZI-M/CT	OLM/HCTZ	AZI-M/CT	OLM/HCTZ	AZI-M/CT	OLM/HCTZ	AZI-M/CT	OLM/HCTZ	AZI-M/CT	OLM/HCTZ	AZI-M/CT	OLM/HCTZ	AZI-M/CT	OLM/HCTZ	AZI-M/CT	OLM/HCTZ	AZI-M/CT	OLM/HCTZ
Cushmann et al. (2012) [5]	20	10	144	34	-	-	57	26	30	15	-	-	16	5	28	8	119	26
Neutel et al. (2017) [11]	21	5	90	36	100.2 (1.19 )	1000.2 4 (1.22)	29	26	23	11	7	2	3	2	13	5	95	60
Cushmann et al. (2018) [12]	5	4	81	25	99.25	99.8	48	29	-	-	-	-	13	5	11	1	101	22
Bakris et al. (2018) [13]	-	-	34	29	-	-	-	-	-	-	-	-	4	3	-	-	3	4

**Figure 5:**
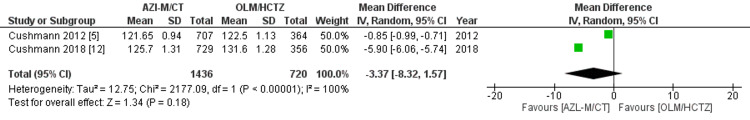
Forest plot of mean SBP. Sources: [5,12]. WMD, weighted mean difference; CI, confidence interval; M-H, Mantel-Haenszel; SBP, systolic blood pressure

**Figure 6:**
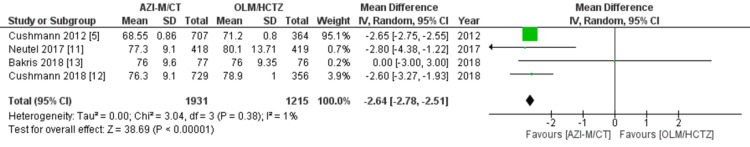
Forest plot of DBP. Sources: [5,11,12,13]. WMD, weighted mean difference; CI, confidence interval; M-H, Mantel-Haenszel; DBP, diastolic blood pressure

**Figure 7:**
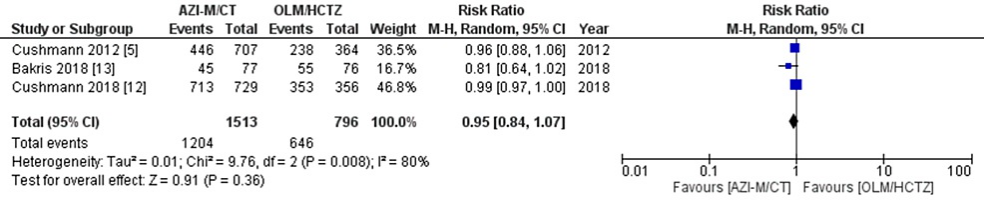
Forest plot of achievement of target blood pressure. Sources: [5,12,13]. CI, confidence interval; M-H, Mantel-Haenszel; IV, inverse variance

Efficacy: The primary outcome measured in this analysis was the change in mean blood pressure at the end of the observation period. Data on average systolic and DBPs were collected from all four studies included in the analysis [5,11,12,13]. The statistical analysis indicated that patients receiving AZI-M/CT had lower SBPs compared to those taking OLM/HCTZ (WMD -2.95 [-6.64, 0.73]; *P *= 0.12; *I*^2^ = 100%), as shown in Figure 5. Moreover, the WMD for DBP was -2.64 (-2.78, -2.51), *P *= 0.00001, and *I*^2^ = 1%, indicating lower values in the AZI-M/CT group, as shown in Figure 6. Additionally, three out of the four studies [5,12,13] reported the number of patients who achieved their target blood pressure. Based on our findings, there was no significant difference between the two groups in terms of reaching their blood pressure goals (risk ratio [RR] 0.95 [0.84, 1.07]; *P *= 0.36; *I*^2^ = 80%), as shown in Figure 7.

**Figure 8:**
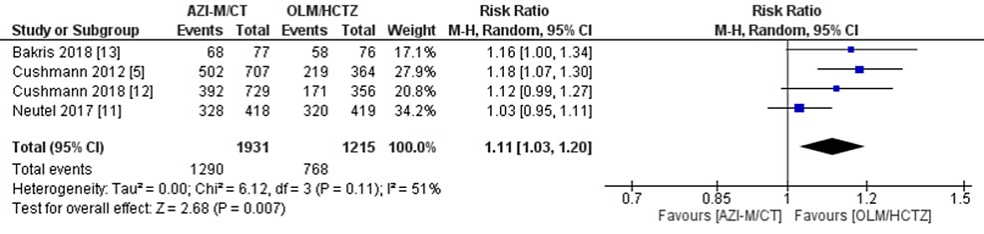
Forest plot of any TEAE. Sources: [5,11-13]. CI, confidence interval; M-H, Mantel-Haenszel; IV, inverse variance; TEAE, treatment-emergent adverse event

**Figure 9:**
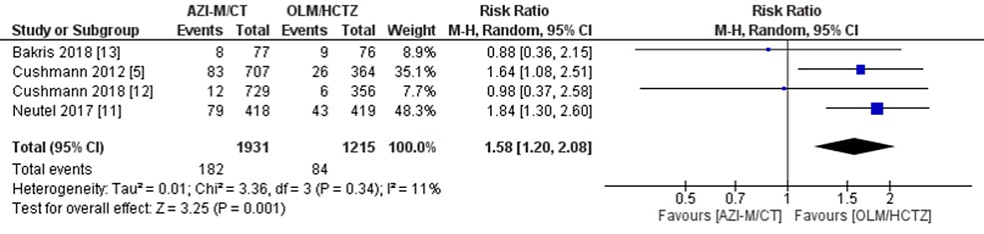
Forest plot of serious adverse event Sources: [5,11-13]. CI, confidence interval; M-H, Mantel-Haenszel; IV, inverse variance

Adverse events: All four investigations by Cushmann et al. [5], Neutel et al. [11], Cushmann et al. [12], and Bakris et al. [13] recorded adverse events. The pooled analysis indicated that patients receiving AZI-M/CT had a higher risk of experiencing any TEAEs compared to those receiving OLM/HCTZ (RR 1.11 [1.03, 1.20]; *P *= 0.007; *I*^2^ = 51%), as shown in Figure 8. Furthermore, the AZI-M/CT group had a significantly increased risk of major adverse events (RR 1.58 [1.20, 2.08]; *P *= 0.001; *I*^2^ = 11%), as indicated in Figure 9. Among the specific adverse events, dizziness showed a statistically significant difference between the AZI-M/CT group and the other groups (RR 1.40 [1.12, 1.74]; *P* = 0.003; *I*^2^ = 43%). However, no significant association was found for headache (RR 0.76 [0.51, 1.14]; *P* = 0.19; *I*^2^ = 43%) and fatigue (RR 1.41 [0.97, 2.04]; *P* = 0.07; *I*^2^ = 0%).

Data on diarrhea and hypotension were available from only two trials by Cushmann et al. [12] and Bakris et al. [13]. The analysis revealed no statistically significant association between the two treatment groups and the occurrence of diarrhea (RR 0.99 [0.10, 9.79]; *P* = 1; *I*^2^ = 74%) or hypotension (RR 1.80 [0.54, 5.97]; *P* = 0.34; *I*^2^ = 0%).

Mortality: Mortality rates were reported in two out of the four studies by Neutel et al. [11] and Bakris et al. [13]. The pooled analysis indicated that there was no significant difference in the mortality rates between individuals treated with AZI-M/CT and those treated with OLM/HCTZ (RR 0.74 [0.14, 3.91]; *P* = 0.72; *I*^2^ = 0%).

Laboratory parameters: Elevated levels of uric acid, potassium, and creatinine were observed in four studies by Cushmann et al. [5], Neutel et al. [11], Cushmann et al. [12], and Bakris et al. [13]. Upon comparing the two treatment groups, our analysis revealed that the AZI-M/CT group had a higher likelihood of experiencing hyperuricemia (RR 1.90 [1.43, 2.53]; *P* = 0.0001; *I*^2^ = 36%) and creatinine elevation (RR 1.79 [1.26, 2.54]; *P* = 0.001; *I*^2^ = 70%). However, there was no significant association between hypokalemia and either treatment group (RR 1.43 [0.78, 2.62]; *P* = 0.24; *I*^2^ = 0%).

The outcomes reported in three of the four investigations by Cushmann et al. [5], Neutel et al. [11], and Cushmann et al. [12] included two consecutive elevations of creatinine (1.5 times the baseline and more significant than the upper limit of normal [ULN]). Our analysis showed no significant association between these elevations and the two treatment groups (RR 1.44 [0.49, 4.26]; *P* = 0.51; *I*^2^ = 73%). However, a shift from normal to low sodium levels was significantly associated with the treatment groups (RR 2.23 [1.24, 4.04]; *P* = 0.008; *I*^2^ = 72%).

Data regarding the shift in fasting glucose from <7.0 to 7.0 mmol/L were available in two studies [5,11]. The results indicated no significant association between the two treatment groups and the shift in fasting glucose from 7.0 to 7.0 mmol/L (RR 1.43 [0.72, 2.88]; *P* = 0.31; *I*^2^ = 55%).

Leave-one-out sensitivity analysis: To address the substantial heterogeneity observed in the pooled analysis of mean SBP and the proportion of patients achieving their goal blood pressure, leave-one-out analysis was performed, This analysis systematically excluded individual studies to assess their influence on the overall results. Notably, the survey by Cushmann et al. [5] had a significant impact on the mean SBP (WMD -4.72 [-6.65, -2.79]; *P* = 0.00001; *I*^2^ = 77%), whereas no single study had a notable impact on the attainment of goal blood pressure.

Discussion

This comprehensive systematic review and meta-analysis, which included four studies involving 3,146 hypertensive patients, aimed to compare the effectiveness of AZI-M/CT and OLM/HCTZ. Both medication regimens achieved the target blood pressure goal of less than 140/90 mmHg (or 130/80 mmHg for patients with diabetes or chronic kidney disease) [14]. Remarkably, AZI-M/CT exhibited greater efficacy than OLM/HCTZ at the same doses, leading to significant reductions in blood pressure across the studies, particularly in DBP. Although SBP reductions were not effective, minor decreases can yield various cardiovascular benefits. For instance, in middle-aged adults, a 2 mmHg reduction in SBP is associated with a 10% reduction in stroke mortality and a 7% decrease in the risk of death from ischemic heart disease [15].

Both treatment regimens were generally well-tolerated, but AZI-M/CT had a higher incidence of TEAE, particularly dizziness. However, most of these side effects were mild to moderate in severity and were more prevalent with higher dosage formulations. Similarly, higher doses of AZI-M/CT were linked to increased rates of significant adverse events (AEs) and discontinuation. It is important to note that many of these discontinuations were likely due to patients being withdrawn from treatment according to protocol guidance, as they experienced elevated serum creatinine levels.

Patients in the AZI-M/CT group exhibited significantly higher creatinine levels. However, these elevations were reversible upon discontinuation of therapy. They reflected a physiological effect of the medications' mechanism rather than a side effect. When ARBs are prescribed to patients with renal disease, a 35% increase in serum creatinine is commonly observed as blood pressure decreases [16]. More significant reductions in blood pressure would lead to greater gains in creatinine, indicating the drug's efficacy [17]. ARBs inhibit the renin-angiotensin-aldosterone axis, which causes vasodilation of the efferent arterioles in the glomeruli, increasing renal blood flow while decreasing the glomerular filtration rate. This results in elevated blood metabolites such as urea and creatinine [17]. Patients with chronic hypertension who have impaired renal blood flow autoregulation due to endothelial dysfunction may be more susceptible to this effect [18]. Concurrent administration of potent diuretics like CLD may further exacerbate creatinine elevation by inducing blood volume contraction [17].

The enhanced efficacy of AZI-M/CT can be attributed to the unique advantages of each component drug. A study by White et al. compared the effects of AZI-M with those of OLM and valsartan (VAL). It revealed that 80 mg of AZI-M resulted in a minor reduction in mean SBP over 24 hours compared to the maximum clinically approved doses of OLM (40 mg) and VAL (320 mg), without a significant increase in adverse effects [19]. AZI-M exhibits a higher affinity for binding to the angiotensin receptor compared to other ARBs, which may contribute to its greater effectiveness [20]. Additionally, AZI-M is more effective than other diuretics, particularly angiotensin-converting enzyme inhibitors (ACE-Is), in lowering blood pressure while having similar or fewer adverse effects, notably the absence of a dry cough. These benefits contribute to improved treatment adherence [20]. CT has a longer half-life than HCTZ, allowing it to maintain its hypertensive efficacy for an extended duration (47-72 versus 16-24 hours) [21]. As a result, CT achieves comparable reductions in office SBP, superior reductions in 24-hour ambulatory BP, and decreased nighttime BP compared to HCTZ [21]. However, a recent observational study found no significant differences between CT and HCTZ regarding cardiovascular outcomes, such as acute myocardial infarction, hospitalized heart failure, and stroke [4]. Furthermore, CT is associated with a significant risk of hypokalemia and other electrolyte abnormalities, making HCTZ the preferred medication. Nevertheless, when CT is combined with an ARB, specifically AZI-M, the incidence of hypokalemia and other electrolyte abnormalities decreases and becomes comparable to OLM/HCTZ [5,13].

Limitations

When interpreting the findings of this meta-analysis, it is essential to consider the limitations associated with the study. First, clinical heterogeneity was observed, which could be attributed to variations in study designs, therapies used, patient's characteristics such as body weight, age, sample sizes, and gender ratios within the patient population, as well as variations in trial characteristics. These factors may have influenced the outcomes and should be considered when concluding. Second, the duration of follow-up varied among most studies, with some reporting more extended follow-up periods. Short-term follow-ups are more useful when assessing the prognosis of a condition. Conversely, long-term follow-ups may present a skewed perspective by indicating either a worsening health deterioration or a more favorable recovery rate. Finally, it should be acknowledged that certain combinations of drugs or doses may not be as effective as others. The effectiveness of treatment may vary depending on the specific drug regimen and dosage used. Overall, these limitations should be considered when interpreting the results of this meta-analysis, and further research is warranted to address these factors and provide a more comprehensive understanding of the efficacy of the interventions under investigation.

This article was previously posted to the Authorea preprint server on November 27, 2022.

## Conclusions

Based on recent systematic reviews and meta-analyses, it has been determined that AZI-M/CT exhibits superior efficacy in reducing blood pressure among elderly patients with hypertension when compared to OLM/HCTZ. However, to substantiate these findings, conducting more extensive clinical studies that specifically compare the effectiveness and safety profiles of AZI-M/CT and OLM/HCTZ is essential. Such studies would provide more robust evidence to support the observed benefits of AZI-M/CT over the alternative treatment option.
